# Analysis of the characteristics of geography study tour program development in China

**DOI:** 10.1371/journal.pone.0330505

**Published:** 2025-09-08

**Authors:** Dinghui Liu, Wentao Yang, Xinrui Zhan, Chuanci Feng

**Affiliations:** College of Urban and Environmental Sciences, Hubei Normal University, Huangshi, China; Zhejiang Agriculture and Forestry University: Zhejiang A and F University, CHINA

## Abstract

Using 730 winning entries in the 2023 “Kyushu Cup” National Study Tour Program Design Competition as examples, this article analyzes characteristics of geography study tour program development in China via GIS and SPSS software. The study results show that the development of geography study tour programs in China is unbalanced in terms of spatial distribution, course content, main body of design, and field trip scope. The following manifestations were observed: first, there are large spatial and administrative differences in the development of Chinese geography study tour programs; second, although geography is a comprehensive discipline that emphasizes the holistic nature of regional studies, the proportion of comprehensive regional study tours is relatively low; third, colleges and universities are the primary developers of geography study tour programs, followed by secondary schools; and finally, the development of Chinese geography study tour programs is dominated by local study tours, which are mainly based on vernacular geography. This study provides new insight into the spatial and administrative disparities in geography study tour programs, highlights the lack of comprehensive regional study tours, and offers practical recommendations for promoting a more balanced and interdisciplinary approach to geography education in China.

## Introduction

The study tour has a long history as an educational practice aimed at learning. In the West, its origins can be traced back to the 16th to 18th centuries in Europe with the rise of the “Grand Tour” movement. During this period, British and German aristocrats traveled to France and Italy to immerse themselves in these regions’ rich history and culture as part of their education [1]. The purpose of the “Grand Tour” movement was to nurture the children of nobility by exposing them to the politics and cultures of other countries, enhancing their refinement, and reinforcing the aristocracy’s rule [2]. In the 19th century, Japan also saw the emergence of study tours, and in 1886, the long-distance expedition of Tokyo Normal High School (now the University of Tsukuba) set a record for a study tour lasting 11 days [3]. In 1958, study tours were officially incorporated into Japanese primary and secondary schools. Since the 1990s, with the implementation of Japan’s international education policy, overseas study tours have gradually become standard practice [4]. In 2006, the UK Department for Education issued the Learning Outside Classroom Manifesto, and in 2010, the UK Parliament recommended that study tours be included in formal national programs [5]. There is a similar Camp Education in the United States. Since 2007, the United States has been promoting the No Child Left Inside Act, a study tour bill that aims to give students more opportunities to learn outdoors by promoting environmental education [6]. In 2012, Barack Obama introduced the America’s Great Outdoors Initiative, aiming to instill in students a sense of nature conservation through outdoor experiences [7]. In 2013, the Chinese government introduced the “National Tourism and Leisure Programme (2013-2020),” outlining plans to gradually introduce study tours for primary and secondary school students. This initiative aimed to enhance students’ self-care skills, creativity, and practical abilities. In 2016, the Ministry of Education of China and 11 other departments issued guidelines on advancing study tours for primary and secondary school students (i.e., “Opinions on Promoting Study Tours for Primary and Secondary School Students”). These guidelines urged local governments to prioritize study tours, fostering their expansion and development. The implementation of these policies has significantly contributed to the growth of educational travel for students across China [8].

Geography study tours are a specialized form of study tour, with geography being well-suited for this approach due to its focus on regions, interdisciplinary connections, and hands-on learning opportunities [9]. The Geographical Association states that fieldwork is central to the study of geography at the primary and undergraduate levels. In the UK, students choosing geography at Level A are required to complete independent fieldwork [10]. Japanese educational authorities regularly organize practical geography field activities for students. These activities include observing and investigating geological features, vegetation, soil characteristics, and aspects of social life. Through these experiences, students develop a deeper appreciation for life, a greater interest in geography, and improved problem-solving skills [11]. Canada is a mecca for geographical studies because of its diverse natural geography and diverse culture. Canadian outdoor educators tend to combine geography and adventure education programs to encourage students to engage in outdoor learning [12]. In 2020, China revamped the Geography Program Standards for General High Schools, noting that study tours are an important learning mode for cultivating core literacy in the discipline of geography and that teachers should guide students to carry out rich and varied practical geography activities in nature, society, and other real-life situations through independent, cooperative, and exploratory learning methods. The new version of the General High School Geography Program Standards encourages the growth of geography study activities in China. However, some local secondary schools face challenges such as limited classroom time, manpower, and financial resources. As a result, study courses remain underdeveloped, leading to ineffective educational outcomes, inadequate support systems, and a lack of well-trained tour instructors, ultimately failing to achieve the goal of practical education for students [13]. The development of high-quality geography study tour programs to meet the growing demand for local study tours has become a popular topic in the education sector. Efforts are increasingly focused on designing and implementing such programs that align with both educational goals and the interests of participants. This trend reflects a growing recognition of the value of immersive, place-based learning experiences in geography education across China.

### Literature review

In recent years, the rapid development of study tours has received widespread attention from the education community and has gradually developed into a compulsory course in primary and secondary education in many countries. Below, this article provides a summary of the key literature on the subject.

#### Grounded theory research.

Basic theoretical research mainly includes the concept, characteristics, and curriculum standards of study tours. Due to different language expressions, there is no standardized definition of a study tour. Some scholars believe that “study tour,” “educational tourism,” “learning travel,” etc., mainly refer to outdoor learning activities with the common purpose of education and tourism, while “outdoor education,” “field trip,” etc., refer mainly to specific teaching methods that are often used in schools and universities to implement experiential education [14]. In general, there is no uniform definition of study tours across countries, and the conceptual scope of outdoor education is considered to be larger than that of study tours, which encompasses both close-range study tours, such as community-based study tours, and far-range study tours with tourist characteristics [15]. Priest defined outdoor education as an umbrella term encompassing all forms of outdoor learning. He identified six key characteristics: it is experiential, takes place outdoors, engages all senses, is interdisciplinary, and focuses on relationships between people and natural resources [16]. China’s Ministry of Education defines study tours in its “Opinions on Promoting Study Tours for Primary and Secondary School Students” as tours that are organized and arranged in a planned manner by education departments and schools and are carried out through group travel that provides centralized accommodations and sustenance as well as out-of-school educational activities combining research studies and travel experiences. Scholars from the Geography Teaching Professional Committee of the China Education Association have compiled the “Study Tour Standards” to promote the standardization, curriculum integration, and quality improvement of study tours. These standards cover the nature and positioning of study tour courses, basic course concepts, and course objectives, structure, content, development, implementation, and evaluation [17].

#### Research on the development of research resources.

In terms of research and development, the study tours mainly involve typical domestic outdoor resources, including parks, museums, monuments, industrial parks, farms, communities, and natural landscapes. For example, in the Muddy Creek Watershed in the eastern part of the Coast Range in the Willamette Valley, Oregon, United States, which includes large areas of national forests and national wildlife refuges, study tours teach local middle school students about the watershed through the development of these natural resources [18]. UK study tours are mainly based on the natural environment and make use of the rich and varied natural landscapes to provide a wide range of possibilities for outdoor activities [19]. Italian educators have a long history of developing resources for study tours. Studio d’ambiente is the main mode of outdoor learning in the country, most commonly including horticultural and agricultural learning [20–21]. China’s vast land area and rich, diverse natural and cultural landscapes provide abundant outdoor resources for tours. In 2017, the Chinese Ministry of Education published a list of 204 national studies and practice education bases for primary and secondary school students. Some local schools have developed local study tour teaching materials. For example, Li Wen Master Teacher’s Studio published the study tour teaching material “Seeking the Beauty of Fuzhou.” This material focuses on eight attractions in Fuzhou and designs a number of study activity routes, guiding students to complete practical activities with geographic tools and methods, recognize and appreciate the geographic environment with geographic vision, and improve their geographic practical and aesthetic abilities [22].

#### Research on the design of study tour programs.

In terms of study tour program design, studies have emphasized the construction of outdoor education practice bases, highlighting students’ roles in field trip activities, adopting problem-based or project-based learning as the teaching mode, and focusing on the use of information technology. For example, Krakowka proposed an innovative model based on secondary school geography study tours that categorized field trips into community field trips to discover details about the region, scavenger hunts to find specific locations in the region based on clues provided by the teacher, and virtual field trips using Google Earth [23]. In the UK, research programs clarify and refine the objectives and tasks of study travel, emphasizing students as the central focus. Teachers guide outdoor activities, integrating thematic knowledge into the outdoor environment [24].

Study tours require students to leave the classroom and conduct a variety of practical activities in authentic natural and social environments. As a result, study tours are interdisciplinary in nature and often linked to curricula such as art, culture, science, geography, physical education, and civic education. [25]. In recent years, cell phones and tablets have become increasingly popular as ideal tools for supporting learning outside the classroom. Medzini et al. found that by using cell phones and tablets during a study tour, it is possible to increase the fun and interactivity of students’ outdoor activities and improve their learning [26].

#### Research on the dilemmas of study tour programs.

Dilemmas with study tours have also attracted the attention of scholars, with Kiviranta et al. noting that the challenges to having an impact in realizing children’s outdoor learning are related to pedagogical prerequisites, including teachers’ abilities to structure and prepare for outdoor activities [27]. Svobodová et al. conducted questionnaires and interviews with stakeholders in Czech schools and found that teachers and parents believed that outdoor education helped students develop valuable skills and knowledge. In contrast, students viewed outdoor education more as a recreational activity and time away from the classroom rather than a learning experience [28]. Su et al. pointed out that some of China’s study tours face specific challenges including low-quality courses, low participation and popularization rates, lack of evaluation mechanisms, safety, and security—all of which impede the goals of acquiring knowledge and skills through outdoor practice [29]. Zhang et al. conducted in-depth interviews with 196 stakeholders in the Chinese tourism sector, primary and secondary schools, study tour service providers, and study tour camps and showed that the development of study tours in China suffers from problems such as a lack of safety and security, poor program quality, weak professional capabilities, and low participation and popularity rates [30]. To address these challenges, scholars have proposed several countermeasures. These include clarifying and refining the objectives and tasks of study tours and their implementation [31], enhancing teacher training in outdoor education skills [32], strengthening risk awareness [33], mobilizing the enthusiasm of all main parties, and forming a collaborative network for outdoor education that involves the government, schools, families, and other parties [34].

In summary, existing research on the development of study tour programs mainly analyzes the development of study tour resources, the design of study tour programs, and existing problems from an educational perspective. There is relatively little research on the regional characteristics and regional differences in the development of study tour programs from a geographical perspective. In terms of research methods, existing studies tend to focus on qualitative analysis rather than quantitative analysis. Quantitative research mainly involves interviewing teachers and students through interviews and questionnaires to reflect the degree of student interest in study tours, the current status of study tour program development, and existing problems. There are still few papers that use information technology to conduct relevant analysis. Existing research has not analyzed the problems in the development of study tour programs in a specific region of China, resulting in a lack of regional specificity in the proposed countermeasures, which makes it difficult to meet the actual needs of study tour program development in different regions.

**Fig 1:**
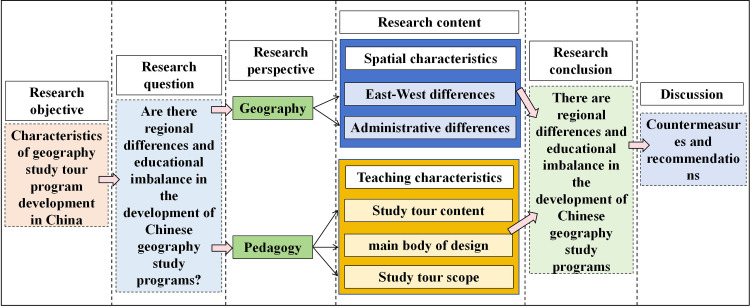
A theoretical and analytical framework for the characteristics of geography study tour program development in China.

Are there regional differences and educational imbalances in the development of study tour programs in China? There is currently a lack of relevant academic research on this issue. Therefore, this paper combines pedagogy and geography, descriptive statistics, and spatial analysis and uses the winning entries of the 2023 Kyushu Cup China National Study Travel Program Design Competition as examples to discuss the characteristics, regional differences, and educational imbalances of the development of geography study tour programs in China using SPSS and GIS software. On this basis, it will make recommendations for the balanced regional development of China’s geography study tour program development in the future (Fig 1). This paper not only provides a new spatial analysis perspective for study tour program development research but also fills the research gap in terms of the spatial differences and educational imbalances in China’s study tour program development.

## Materials and methods

### Data sources and processing

Following the joint issuance of a document by the Ministry of Education and 11 other departments in 2016, a surge in study travel has occurred across China. Primary and secondary schools have increasingly relied on their own resources and social forces to organize and conduct a variety of study tour practices. The practical, regional, and living characteristics of geography are highly compatible with the educational and regional characteristics of tourism. This makes geography the leading course in the school’s integrated practical activity program and the most suitable course for conducting study tours. Since 2020, the organizing committee of the Kyushu Cup Study Tournament, guided by three major geography education academic journals—“Secondary School Geography Teaching Reference,” “Geography Teaching,” and “Geography Education”—in collaboration with a group of academic education journals, has undertaken a series of large-scale national study tours and competitive academic events, yielding notable achievements and an extensive impact. The online 2023 Kyushu Cup National Study Tour Program Design Competition, held from March to June, witnessed an unprecedented number of participating schools, a substantial increase in the number of submissions—receiving over 1,100 submissions from more than 260 units (including over 130 colleges and universities, over 120 primary and secondary schools, and over 10 related study organizations) from across the country—and a notable enhancement in their quality. Of the entries, 730 were awarded prizes. This article uses these winning entries as a representative sample of study tours and analyses the characteristics of China’s geography study tour program development by using GIS and SPSS software.

**Fig 2:**
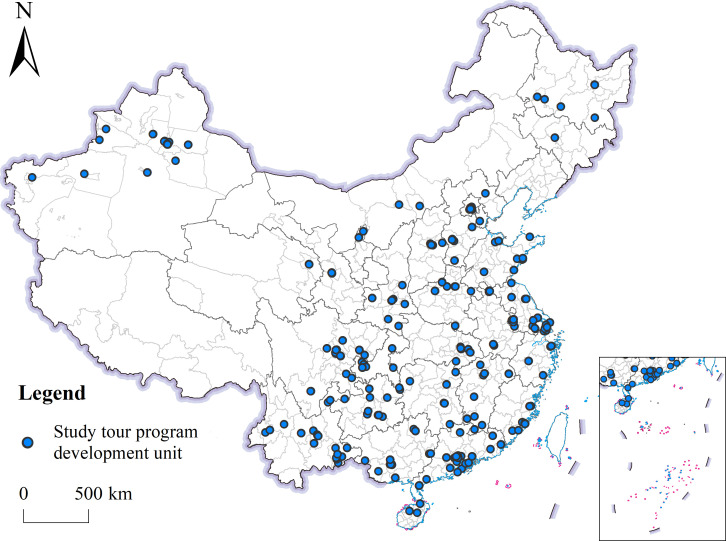
Distribution of geography study tour program development units in China.

The data used in this study were obtained from the official records of the Kyushu Cup National Study Tour Program Design Competition (https://mp.weixin.qq.com/s/-943Z1egImBeLCXpZG4AUw). No ethical approval was required for this study. We first converted the address of each awarded unit into geographic coordinates and then imported the geographic coordinates into ArcGIS 10.8 software for data analysis. Fig 2 provides a spatial visualization of this ArcGIS transformation of the study tour program awardee data.

A map of China’s administrative regions was obtained from the Standard Map Service website of the Ministry of Natural Resources (http://bzdt.ch.mnr.gov.cn/index.html).

### Data analysis

#### Kernel density estimation (KDE).

Kernel density estimation (KDE) is used to describe the denseness of the distribution of geography research program development units in the study area. The KDE can calculate the degree of data clustering and dispersion in the entire area based on the sample data, and a higher density value indicates that the samples are more spatially clustered. Points in the search radius have different weights, and the closer to the center of the search circle, the greater the weight given. Finally, we obtain a continuous density surface, which is calculated as [35]:


$$\int_{n}(x)=\frac{1}{nh}\sum_{i=1}^{n}k(\frac{x-x_{i}}{h})$$ (1)

where k is the kernel function, h is the search radius, and n is the number of sample points, the result of which is the kernel density of point x. In actual calculations, the size of the search radius h will have different effects on the results: if the value of h is too small, the kernel density map will not be sufficiently smooth, and the overall picture will be too abrupt; if the value of h is too large, some of the real details of the calculation results will be obscured. Through multiple comparative tests, it was found that 30 km can better reflect the density center of the distribution of geography study tour development units.

#### Standard deviational ellipse.

A standard deviational ellipse was used to measure the directionality of the sample distribution and consisted of three main elements: corner, standard deviation along the long axis, and standard deviation along the short axis. The direction of the distribution of the long axis of the ellipse represents the direction of the largest spatial distribution of samples, and the short axis represents the direction of the smallest spatial distribution of samples [36]. A standard deviational ellipse was used to reflect the directionality of the spatial distribution of the development units of China’s geography study tour programs. The calculation formula is as follows:


$$E_{x}=\sqrt{\frac{\sum_{i=1}^{x}\left(x_{i}-\overset{―}{X}\right)}{n}}$$ (2)

$$E_{y}=\sqrt{\frac{\sum_{i=1}^{y}{(y}_{i}-\overset{―}{Y})2}{n}}$$ (3)

$$\text{tan}\theta =\frac{\left(\sum_{i=1}^{n}a_{i}^{2}-\sum_{i=1}^{n}b_{i}^{2}\right)+\sqrt{{\left(\sum_{i=1}^{n}a_{i}^{2}-\sum_{i=1}^{n}b_{i}^{2}\right)}^{2}+4{\left(\sum_{i=1}^{n}a_{i}b_{i}\right)}^{2}}}{2\sum_{i=1}^{n}a_{i}b_{i}}$$ (4)

where E_x_ and E_y_ are the major and minor axes of the standard ellipse, X_i_ and Y_i_ are the coordinates of element i, Y is the average center of all elements, n is the number of elements, θ is the rotation angle of the standard ellipse, and a_i_ and b_i_ are the distances from element i to the average center in the major and minor axis directions.

#### Hu Huanyong line.

The “Hu Huanyong Line” is the boundary of the population density distribution in China, as proposed by Hu Huanyong, a famous Chinese population geographer. The line runs from Aihui County (now Heihe City) in Heilongjiang Province in the east to Tengchong City in Yunnan Province in the west; it is a straight line running northeast to southwest, dividing China into two major sectors: the densely populated southeast and the sparsely populated northwest. This line has attracted much attention since its inception, not only becoming an important baseline for China’s population geography but also profoundly influencing the definition and perception of China’s economic, social, and cultural spatial patterns [37]. In this study, we use the Hu Huanyong Line as a demarcation line to reveal the spatial imbalance in the development of geography study programs in China.

## Results

### Spatial differences in the development of geography study tour programs in China

**Fig 3:**
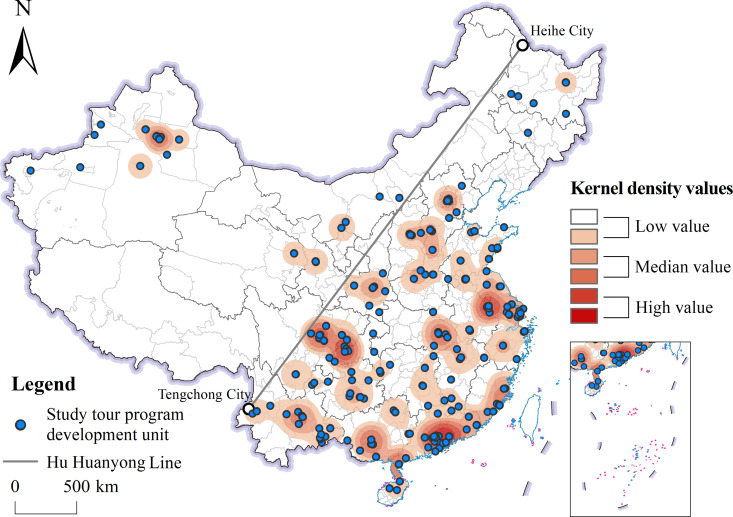
Kernel density analysis of geography study tour program development units in China.

ArcGIS 10.8 software was used to convert the participating award-winning units into point coordinates and to analyze the kernel density (Fig 3). As shown in Fig 3, the distribution of award-winning units participating in the Kyushu Cup National Study Tour Program Design Competition shows a clear east–west spatial difference. Taking the Hu Huanyong Line as the boundary, there were 242 award-winning units in the area east of the Hu Huanyong Line accounting for 90.3% of the total number of award-winning units, while there were only 26 award-winning units in the area west of the Hu Huanyong Line, accounting for only 9.7%. The density of the distribution of award-winning units was analyzed using the KDE method, and the results showed that the main density cores of award-winning units were concentrated in the Pearl River Delta, Yangtze River Delta, and Chengdu-Chongqing regions, while the secondary density cores were concentrated in Wuhan, Kunming, Nanning, Urumqi, Xi’an, Beijing, Shijiazhuang, Taiyuan, and other cities. The overall distribution shows a spatial pattern of “more in the east and less in the west, more in the south and less in the north.” It can be seen that the spatial distribution characteristics of China’s geography study tour program development are basically consistent with the spatial distribution characteristics of China’s population. With the Hu Huanyong Line as the boundary, the population of the northwestern half of China accounts for about 6% of the total population, while that of the southeastern half accounts for about 94%. This unbalanced population distribution pattern has remained basically unchanged for more than half a century. The western region is constrained by natural environmental factors such as terrain and precipitation, resulting in significant internal variations in population distribution. Urban agglomerations centered on provincial capitals have become regions of population concentration [38]. Additionally, there are significant disparities in economic and educational resources on either side of the Hu Huanyong Line. Over the past 20 years, the northwest half has accounted for about 5% of the GDP and 7% of educational resources, while the southeast half has accounted for about 95% of the GDP and 93% of educational resources [39]. The east–west imbalance in the distribution of China’s population, economy, and educational resources has led to significant east–west differences in the development of geography study tour programs. The eastern region is economically developed, has a strong teaching force, and has more educational resources and capital investment, which provides a good material foundation and conditions for the development of geography study tour programs. In contrast, due to the relative scarcity of economic and educational resources in the western region, the development of geography study tour programs has been somewhat limited. Although the western region also has unique natural and cultural landscapes, these advantageous resources have not been fully utilized and developed due to a lack of sufficient capital and teaching resources.

**Fig 4:**
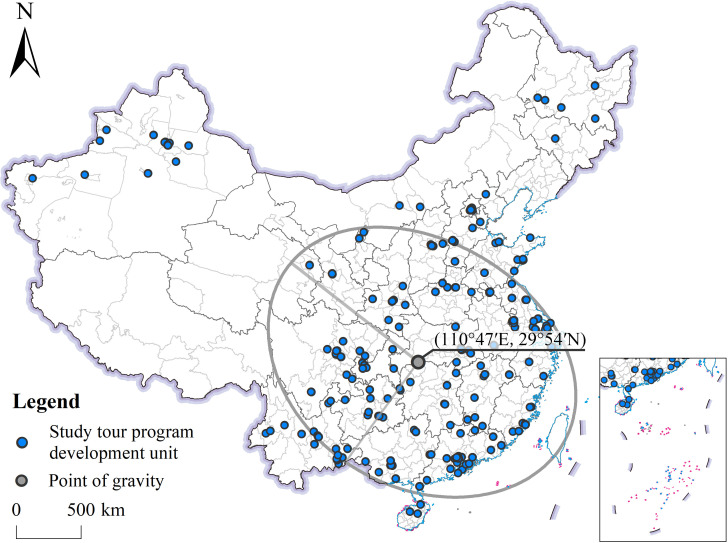
Standard deviational ellipse of Chinese geography study tour program development units.

The directionality of the spatial distribution of award-winning units was measured using a standard deviational ellipse, with the direction of the long axis of the ellipse representing the direction of the largest spatial distribution of award-winning units (Fig 4). As can be seen from Fig 4, the award-winning units as a whole tend to be concentrated in the “northwest–southeast” direction, with the center of distribution being (110°47′E, 29°54′N) in Changyang Tujia Autonomous County, Yichang City, Hubei Province. This distribution direction is consistent with the distribution direction of regions with high award densities, such as the Pearl River Delta, Chengdu-Chongqing, Wuhan, and Urumqi. On one hand, this indicates a high degree of geography study tour program development in these regions; on the other hand, it also indicates that the development of geography study tour programs in regions such as Northeast China needs to be strengthened. This is related to the influence of competition. The organizers of the competition, the editorial offices of the three major academic journals of geography education, are located in Xi’an, Shanghai and Chongqing, which are far away from Northeast China and North China, resulting in minimal influence of the competition in these regions, to a certain extent, which affects the motivation of the region to participate in the competition.

### Administrative differences in the development of geography study tour programs in China

**Fig 5:**
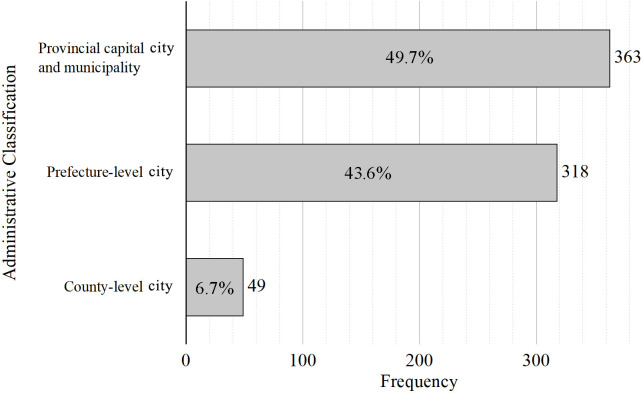
Administrative classification of the location of geographic research and development units in China.

Statistics on the administrative hierarchy of the location of the study tour program development units showed that nearly half of the units were in provincial capital cities and municipalities directly under the central government, 43.6% were in prefecture-level cities, and only 6.7% were in county-level cities (Fig 5). This reflects the fact that the development of geography study tour programs in China has obvious hierarchical administrative differences in space. Universities and secondary schools are more active in developing geography research courses in economically developed provincial capital cities and municipalities with higher education levels. County-level cities, where educational resources are limited and information access is restricted, face challenges in developing geography study tour programs. Local schools often do not prioritize these programs, resulting in a significant gap between the development of study tours in these areas and those in provincial capitals, municipalities, and larger cities.

### Characteristics of the content development of geography study tour programs in China

**Fig 6:**
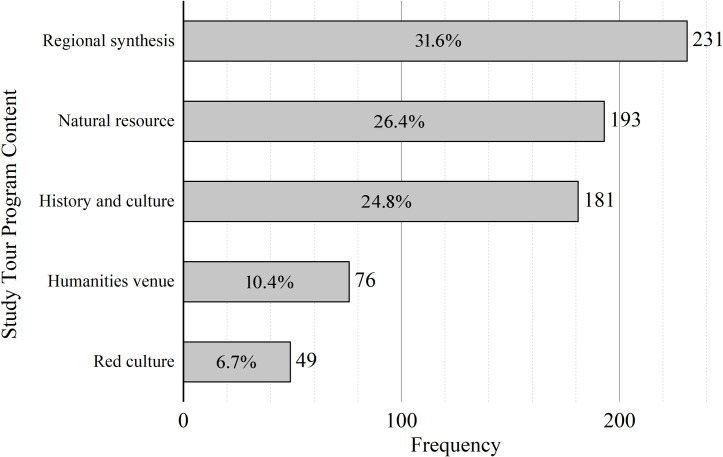
Classification of Chinese geography study tour program contents.

From a scientific perspective, classification is the foundation of all research, including that of study of study tours. In this study, we classify according to the tenets of Geography Study Tour Program Design and Cases, edited by Duan et al., categorizing the contents of the geography study tour into five categories: natural resources, history and culture, humanities venues, red culture, and regional synthesis [40]. Among them, study tour programs centered on natural resources mainly consider natural landscapes such as plants, animals, soil, terrain, geology, and water resources as study objects; history and culture study tour programs mainly consider cultural landscapes such as relics of ruins and local customs as study objects; study tour programs centered on humanities venues mainly consider museums, exhibition halls, industrial parks, and other places as study objects; red culture study tour programs mainly consider war relics, war memorials, martyrs’ cemeteries, and other resources that promote the spirit of patriotism as study objects; and regional synthesis study tour programs consider the typical natural landscape and cultural landscape of regions as study objects. Fig 6 demonstrates this categorization of the content of award-winning programs.

As seen in Fig 6, in China’s geography study tour programs, the proportion of regional synthesis study tours is the highest, accounting for approximately 31.6%, followed by natural resources and history and culture study tours, accounting for 26.4% and 24.8%, respectively, while humanities venues and red culture study tours account for a relatively small proportion, at only 10.4% and 6.7%, respectively. The geography curriculum in schools mainly contains modules of natural geography, human geography, and regional development. The results show that the contents of geography study tour programs, geography textbooks, and regional study tour resources are highly compatible. Geography is a comprehensive discipline that takes human–earth relations as its object of study and emphasizes the holistic nature of regional studies; however, the proportion of comprehensive regional study tour programs is relatively low, accounting for only about 31.6%.

### The imbalance in the development of geography study tour programs in China

**Fig 7:**
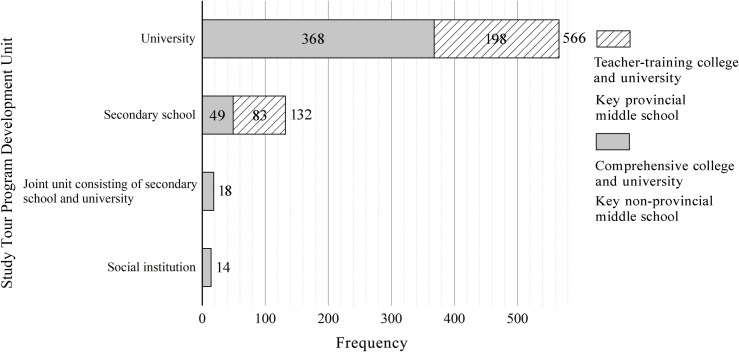
Classification of geography study tour program development units in China.

The units with award-winning work were categorized into four types: secondary schools, universities, social institutions, and joint units consisting of secondary schools and universities. It was found that the highest proportion of geography study tour program development units were universities, accounting for 77.5%, followed by secondary schools, accounting for only 18.1%; the proportions of social institutions and joint units consisting of secondary schools and universities were smaller, accounting for 1.9% and 2.5%, respectively (Fig 7). It can be seen that most geography study tour program development occurs at colleges and universities, followed by secondary schools. Among them, teacher-training colleges and universities accounted for 65%, comprehensive colleges and universities for 35%, key provincial middle schools for 37.1%, and key non-provincial middle schools for 62.9%. There are clear regulations and requirements for field-practice classes in personnel training programs for geography majors in higher education institutions, and universities allocate funds for professional internships. Colleges and universities, with their strong teaching staff and more flexible class schedules, have become the primary institutions driving the development of geography study courses in China. In contrast, secondary schools face challenges such as limited class time, funds, and experienced teachers for field internships, making them less equipped than higher education institutions to develop and implement geography study tour programs.

### The imbalance of the scope of the development of geography study tour programs in China

According to whether the study tour sites were outside provincial boundaries, geography study tours were divided into local study tours—based on vernacular geography in the province—and offsite study tours—based outside the province. A cross-tabulation analysis of the study tour sites and units was carried out using SPSS software (Table 1). The row variable is the study tour unit, which is divided into four categories: secondary schools, colleges and universities, social institutions, and joint units. The column variable is the study tour site, which is divided into local study tour and off-site study tour. The analysis results show that Pearson chi-square = 18.06, with a significance level P-value < 0.001. This indicates that study tour units have a significant influence on the selection of study tour sites; that is, different types of study tour units will choose study tour locations within different ranges. As shown in Table 1, the development of geography study tour programs in China is based on local geography, accounting for 80.4%, while off-site study tour programs account for only 19.6%. Local study tours that focus on vernacular geography have the advantages of proximity, short duration, low cost, and familiarity with the study environment and content. Although both secondary schools and colleges use local study tours as the main objective of field trips, the proportion of local geography studies in secondary schools is much higher than in colleges, reaching 92.4%. Secondary schools often choose locations close to the school for geography study tours, which are shorter in duration, considering the constraints of class time, management, and safety, whereas colleges and universities have fewer constraints and can conduct long-distance geography study tours, which can last up to a week.

**Table 1:** Cross-tabulation analysis of study tour sites and units.

Study tour units		Local study tour	Off-site study tour	Total
Secondary schools	Count	122	10	132
	Percentage	92.4%	7.6%	100%
Colleges and universities	Count	437	129	566
	Percentage	77.2%	22.8%	100%
Social institutions	Count	11	3	14
	Percentage	78.6%	21.4%	100%
Joint units consisting of secondary schools and universities.	Count	17	1	18
	Percentage	94.4%	5.6%	100%
Total	Count	587	143	730
	Percentage	80.4%	19.6%	100%

Pearson chi-square value=18.06, df=3, P<0.001

## Discussion

Using 730 winning entries of the 2023 Kyushu Cup National Study Tour Program Design Competition as examples, this article analyses characteristics of geography study tour program development in China using GIS and SPSS software. The results revealed that the development of geography study tour programs in China is unbalanced in terms of spatial distribution, course content, main body of design, and field trip scope. Based on the above analysis, this study proposes the following suggestions for the balanced advancement of geography study tour program development in China.

### Improvement of regional balance in the development of geography study tour programs

As previously mentioned, large spatial and administrative differences exist in the development of Chinese geography study tour programs. This is reflected in the spatial distribution pattern of “more in the east and less in the west, more in the south and less in the north” and in the imbalance in distribution among administrative levels. From the analysis, it is clear that there is a significant gap in the development of geography study tour programs in regions such as Tibet, Qinghai, Gansu, Inner Mongolia, western Sichuan, and northeastern China, where geography study tours are not given sufficient attention. However, most of these regions have rich resources for geography study tours, so the on-site Kyushu Cup National Study Tour Program Design Competition can be held in these regions. Thus, through the organization and publicity of the competition, local geography teachers can be invited to observe and learn from the competition, so as to help them improve the level of the development of geography study tour programs in the local area.

Regarding the problem of administrative differences in the development of study tour programs, it is possible to strengthen exchanges and cooperation in the development of study tour programs among regions with different administrative levels to permit sharing of study tour program resources. Additionally, joint provincial internships can be organized during the summer and winter vacations, allowing geography teachers from county-level schools to collaborate with experienced master’s-level educators in research. This initiative would support regions with less advanced educational resources in enhancing their capacity and motivation to develop geography study tour programs, fostering diverse, distinctive approaches tailored to their unique contexts.

### Focus on the comprehensive development of geography study tour program content

The content of geography study tours refers to the collection of resources such as the natural environment, human history, folklore, and art within a certain region that is transformed by educators to achieve the goal of educating people and is characterized by distinctive regionality and comprehensiveness. Therefore, the development of geographical study tour content should be more comprehensive. This comprehensiveness has two implications. First, it emphasizes the synthesis of geography, that is, to highlight the human–earth relationship in the geographical system. Geographical systems of human-earth relations refer to human activities and the geographic environment in a specific geographical area formed by mutual constraints and an interdependence system. In the human–earth relationship geographic system, human activities and the geographic environment interact with each other, and neither party can exist in isolation. Geography study tours cannot completely separate natural geography and human geography,–“one side of the soil raises one side of the people,”–and only by highlighting the main line of human–earth relations can the regional and comprehensive nature of geography be manifested in geography study tours.

Second, comprehensiveness refers to interdisciplinary thematic learning in geography study tours. Geography study tours are educational activities conducted in real geographic environments, where participants encounter practical problems that cannot be solved using only the knowledge and methods of geography. Therefore, this articles proposes a geography study tour program based on interdisciplinary learning. Such learning is a form of integrated education based on students’ foundations, experiences, and interests, centered on a research theme, with the content of geography programs as the backbone, and uses and integrates relevant knowledge and methods from other programs. In the process of geography study tours, the programs should not only focus on the learning of geographic knowledge and skills but also integrate the knowledge and skills of other disciplines in the context of the actual situation, guide students to mobilize multidisciplinary knowledge to solve real regional problems, and cultivate students’ comprehensive thinking.

### Strengthening cooperation among the main bodies of research and study program development

Since the National Tourism and Leisure Outline (2013–2020) issued by the General Office of the State Council in 2013 first proposed the idea of gradually implementing study tours for primary and secondary school students, many documents on study tours have been issued by the education department and study tours have become an important initiative in China’s secondary school education reform. However, as shown in the analysis above, universities, not secondary schools, are the main drivers of geography study tour program development in China. This indicates that the current development of geography study programs does not meet the demand for such tours in secondary schools, leading to issues such as “travel without learning” and “tourism without research” in many secondary school study tours. In some areas, study tours have shifted into traditional tourism activities that fail to foster students’ core geography literacy, ethical values, and humanistic understanding.

Colleges and universities have a greater advantage in conducting geography study tours. They benefit from a team of experts and scholars with strong professional backgrounds and extensive experience, leading to more mature and comprehensive study course development. Therefore, secondary schools should cooperate with colleges and universities to hire teachers to guide the development of geography study tour programs. Some award-winning works in the study tour program design competition were designed by joint units formed by secondary schools, colleges, and universities; however, the proportion was small, accounting for only 2.5%. In the future, secondary schools should actively strengthen their cooperation with colleges and universities to cultivate renowned geography study tour tutors.

Although the study offers fruitful suggestions to improve geography study tour development, the results are bound by some limitations. For example, given that the study tour units in our analysis were all winning entries of the 2023 Kyushu Cup National Study Tour Program Design Competition there may be a sample bias problem. The competition is jointly organized by three influential academic journals of geography education in China and has successfully held many large-scale national study travel activities and competitions, attracting the participation of secondary school geography teachers and university geography majors and expanding the scale of the competition annually. However, as the organizer of this competition is neither a national government department nor a national-level academic organization of geography majors, such as the Chinese Geographic Society, this has led to its limited influence and authority. Many secondary schools and universities do not recognize the Kyushu Cup National Study Tour Program Design Competition as a national geography competition, which, to some extent, affects the enthusiasm of secondary school geography teachers and university geography majors and may hamper participation. Some excellent geography teachers did not participate in this competition for various reasons, which biased the data analysis. This problem is expected to be resolved as the Kyushu Cup National Study Tour Program Design Competition continues to expand.

## Conclusion

This article analyses characteristics of geography study tour program development in China using GIS and SPSS software. The results revealed that the development of geography study tour programs in China is unbalanced in terms of spatial distribution, course content, main body of design, and field trip scope. The following specific manifestations were observed: first, there are large spatial and administrative differences in the development of Chinese geography study tour programs; second, geography is a comprehensive discipline that emphasizes the holistic nature of regional studies, but statistically, the proportion of comprehensive regional study tours is relatively low; third, the key developers of geography study tour programs are colleges and universities, followed by secondary schools; and finally, the development of Chinese geography study tour programs is dominated by local study tours, which are mainly based on vernacular geography. Based on the research findings, there are significant differences between eastern and western China in terms of the development of geography study tour programs. To promote the balanced development of geography study tour programs in China, it is necessary to increase investment and support in western China to improve the level of education and teaching staff in the region. At the same time, it is also necessary to strengthen exchanges and cooperation between the eastern and western regions, share high-quality educational resources and experiences, and promote the continuous innovation and development of geography study tour programs. Only in this way can the comprehensive and balanced development of geography study tour programs in China be achieved, providing high-quality study tour education services to more students. How to strengthen the development of study tour programs in western regions will be a topic that needs to be studied in depth in the future.

Compared with previous studies that analyzed the development of study tour programs solely from a teaching perspective, this paper combines geographical and educational perspectives to explore the characteristics of the development of geographical study tour programs in China, revealing their spatial differences and educational imbalances, which is of great significance for promoting the popularization of study tour tourism and improving its educational quality. Through a comparative analysis of the development of study tour programs in different regions of China, it is possible to reveal the commonalities and differences among regions in terms of study tour resource development, program design, and implementation effectiveness, providing a scientific basis for the formulation of more targeted study tour program development strategies. These studies are of great significance for promoting the sustainable development of study tours in China.
